# Is fluid resuscitation the “Keyser Soze” of acute kidney injury in trauma patients?

**DOI:** 10.1186/s13054-019-2333-9

**Published:** 2019-02-08

**Authors:** Matthieu Jamme, Omar Ben Hadj Salem

**Affiliations:** 1 Intensive Care Unit, Poissy Saint Germain Hospital, 9-10 rue du champ Gaillard, 78300 Poissy, France; 2 INSERM U-1018, CESP, Team 5 (EpReC, Renal and Cardiovascular Epidemiology), UVSQ, Villejuif, France; 3 French Intensive Renal Network (FIRN), Poissy, France

Dear Editor,

We read with great interest the study of Harrois et al. [1] that assessed the prevalence and risk factors of acute kidney injury (AKI) in trauma patients. In this large cohort, AKI was frequently observed, especially in patients admitted with hemorrhagic shock (42.5%) and was associated with hypoperfusion markers (blood lactate, minimum prehospital mean arterial pressure, maximum prehospital heart rate) and severity of trauma (hemorrhagic shock and higher Injury Severity Score). Interestingly, patients who required vasopressors did not develop more AKI, suggesting that in addition to renal ischemia, many different physiopathological pathways may be involved in AKI.

Due to these results, the authors have logically suggested systemic inflammation, rhabdomyolysis, renal parenchyma injuries, and ischemic tubulopathy as possible mechanisms leading to AKI. However, information concerning fluid administration during prehospital and hospital periods were not mentioned.

Although the use of colloids was recently restricted in the last European guidelines, due to hemostatic effects, for the benefit of crystalloids solutions [2], it is now well known that colloids are also nephrotoxic due to an accumulation of lysosomes in proximal tubular cells, also called osmotic nephrosis [3]. However, an excess of 0.9% NaCl solution administration causes hyperchloremia, a powerful vasoconstrictor agent of glomerular afferent arterioles and, i.e., a potential factor to extend renal ischemia [4]. Consequently, guidelines suggest to not use more than 1500 mL of 0.9% NaCl solution [2]. In case of head trauma, in which hypotonic solution should be avoided, fluid resuscitation becomes tricky.

Several promising studies had recently identified new potential therapeutic agents of ischemic tubulopathy [5] or rhabdomyolysis, which highlight the need for early identification of mechanisms underlying AKI.

In their report, the authors did not provide data concerning the volume of crystalloid administration, volume of colloid perfused, and indirect biomarker of a high infusion of crystalloid (high base deficit, hyperchloremia) or colloid (hypocalcemia). We hypothesize that these variables are possibly implicated with AKI following major bleeding.
